# Supplementary material for: Emergence of a field-driven U(1) spin liquid in the Kitaev honeycomb model
Source: Nat Commun. 2019 Jan 31;10:530. doi: 10.1038/s41467-019-08459-9 (PMC6355955; doi:10.1038/s41467-019-08459-9)
Supplement: Supplementary file 1 — Supplementary Information [file 41467_2019_8459_MOESM1_ESM.pdf]

Supplementary Information
Emergence of a Field-Driven $U(1)$ Spin Liquid in the Kitaev Honeycomb Model

Ciarán Hickey and Simon Trebst
Institute for Theoretical Physics, University of Cologne, 50937 Cologne, Germany

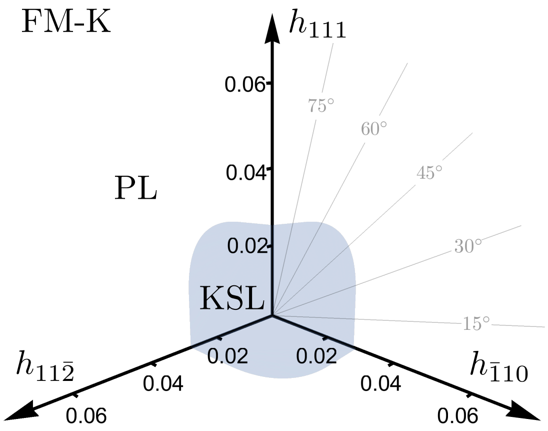

Supplementary Figure 1. Phase diagram in uniform magnetic field. For the pure FM Kitaev model, shown here, there are just two phases, the KSL and PL phases, and a single transition between them.

SUPPLEMENTARY NOTE 1: FERROMAGNETIC KITAEV PHASE DIAGRAM

For ferromagnetic Kitaev exchange the phase diagram in tilted magnetic fields exhibits just two phases, the gapped KSL and the trivial PL phase. The phase diagram is shown in the main text in Fig. 1(b). In Supplementary Figure 1 we provide the same phase diagram, but with the axes scales reduced by an order of magnitude so as to make the boundaries of the KSL phase more visible.

SUPPLEMENTARY NOTE 2: DIRECT KSL-PL TRANSITION

Along certain field directions there is a direct transition from the KSL to the PL phase, with no intermediate GSL phase. In Supplementary Figure 2 we show a selection of data for such a scenario. In particular we take a cut at $30°$ away from the $[\bar{1}10]$ direction, within the honeycomb plane, toward the $[111]$ direction, perpendicular to the plane. We show the energy spectrum, the dynamical structure factor for the Γ' point and the specific heat as a function of increasing field magnitude.

SUPPLEMENTARY NOTE 3: DYNAMICAL STRUCTURE FACTOR

In the main text, it has been argued that the flux gap at zero field breaks apart and moves to lower energy as the external magnetic field is increased, eventually closing fully as the transition to the GSL is crossed. As an example we showcased, in Fig. 4(d) of the main manuscript, the intensity at the Γ' point as a function of field magnitude, with two transitions clearly visible as the spin gap closes at the KSL-GSL transition, remains closed throughout the GSL phase, and then reopens again at the GSL-PL transition. Here we show similar plots for other high-symmetry points, namely examples of M, X and K points. At all of these momenta the same trend is undoubtedly present, with the flux gap at zero field being broken apart and physical spin spectral weight being pushed down to zero energy as the GSL is entered.

However we note that, depending on the field direction, this trend may not be true for all momenta. In particular, if the field along a particular spin axis is negligible, the points in momentum space associated with the corresponding spin component will not be as strongly affected (as spin and momentum space are intrinsically linked together in the Kitaev model, for example the bond in real space associated with say the x-component of the spin translates into a particular direction in the BZ). As a result the flux gap will only fully collapse for those momenta along which the corresponding field magnitude is large. An example of this is shown in Fig.Supplementary Figure 4 where we plot the dynamical spin structure factor in the middle of the GSL along the cut at $\theta = 7.5°$ away from the $[\bar{1}10]$ axis. In this case the field along the z-axis, h_z, is much smaller compared to the other two components. This results in the flux gap remaining mostly intact at, say, X_1 and K_1 for example, while it closes at the remaining momenta. This raises the interesting possibility that the gauge field excitations may be anisotropic. In other words the gauge field propagator may be gapless for excitations with $\vec{k}$ along certain directions, and gapped for other directions, which could be captured by a $\vec{k}$-dependent mass.

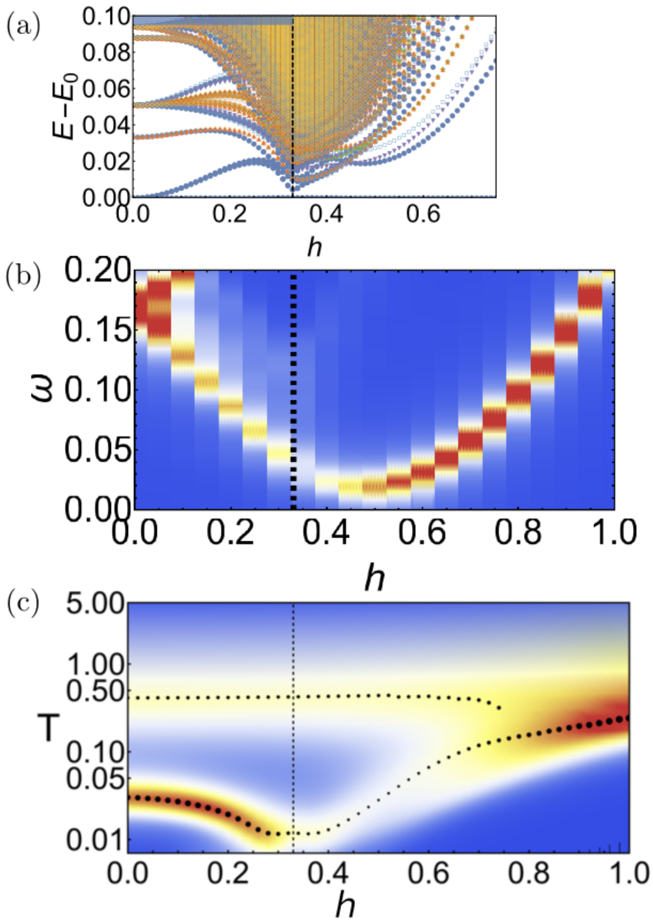

Supplementary Figure 2. Direct KSL to PL transition. Data with (a) the energy spectrum, (b) the dynamical spin structure factor at the Γ' point, and (c) the specific heat shown for a cut $30°$ away from $[\bar{1}10]$ toward $[111]$.

SUPPLEMENTARY NOTE 4: STABILITY OF THE FERMI SURFACE

Regarding the stability of the Fermi surface, though a normal Fermi liquid is unstable to an infinitesimal attractive interaction, coupling to a dynamical $U(1)$ gauge field stabilizes the Fermi surface up to a finite critical strength of the interaction [1]. Further, we note that while there are several symmetry-allowed fermionic pairing terms, these will carry a non-trivial momentum dependence due to the shift of the Dirac cone away from high-symmetry momenta for generic field directions and as such will not contribute to the low-energy sector, as at least one of the momenta is far away from the Fermi surface. Note, however, that for magnetic fields applied along high-symmetry directions, some of these pairing terms might become relevant and thereby lead to a spin-Peierls instability akin to what has been discussed for Majorana Fermi surfaces [2].

SUPPLEMENTARY NOTE 5: EXACT DIAGONALIZATION RESULTS IN CONTEXT

For those that may not necessarily be familiar with the energy scales typically encountered in exact diagonalization (ED) studies we would like to highlight the smallness of the gaps encountered in this work when compared to some known ED results. In particular we can compare to:

- The regular Heisenberg antiferromagnet on square, triangular and honeycomb lattices. In all cases the ground state is magnetically ordered with gapless Goldstone modes. Examples of spin gaps from ED include, $\Delta = 0.288$ for an $N = 36$ site symmetric square cluster, $\Delta = 0.123$ for an $N = 36$ site symmetric triangular cluster and $\Delta = 0.214$ for an $N = 32$ site symmetric honeycomb cluster. Even the reported extrapolated gaps, taken from considering a range of system sizes, are much larger than those in the GSL (for all cluster sizes we have studied), being $\Delta_\infty = 0.025$ for the square lattice (taken from 12 lattices ranging from $N = 18-40$), $\Delta_\infty = 0.129$ for the triangular lattice (taken from 12 lattices ranging

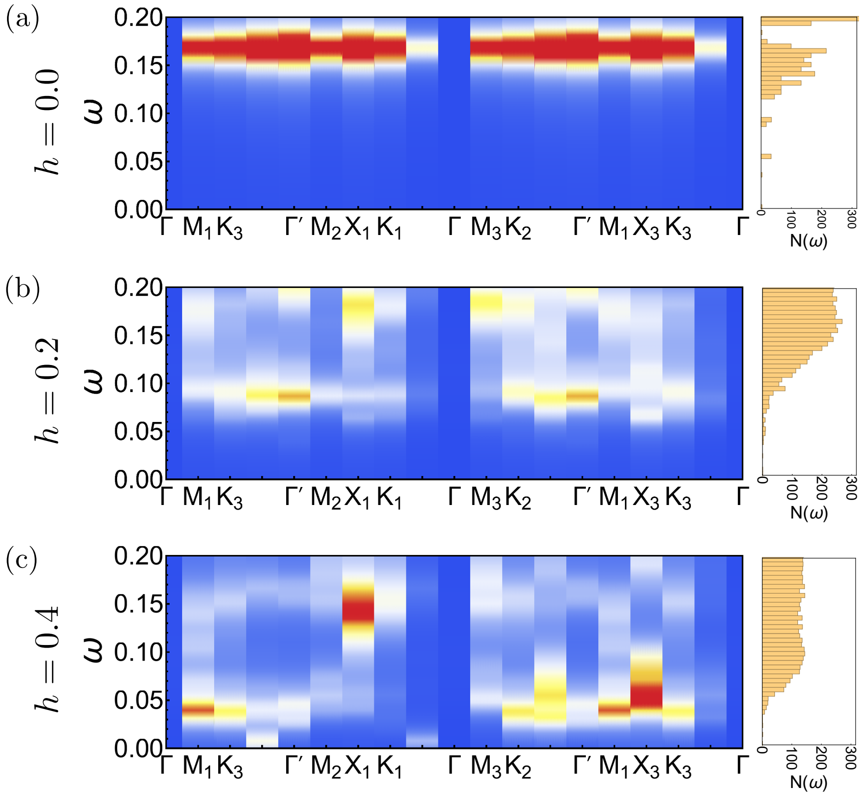

Supplementary Figure 3. Dynamical spin structure factor. (a) The zero-field KSL, (b) a point midway in the KSL phase and (c) a point in the middle of the GSL along a path through all high-symmetry points of the extended Brillouin zone for the cut $\theta = 7.5°$, the lower of the two dashed red lines in Fig. 1(c) of the main text.

from $N = 24 - 36$) and $\Delta_\infty = 0.050$ for the honeycomb lattice (taken from 14 lattices ranging from $N = 6 - 38$). All values are taken from [3] and references therein.

- The Kagome lattice Heisenberg antiferromagnet (KHAFM). There is an ongoing debate as to whether this is a gapless Dirac spin liquid or a gapped $\mathbb{Z}_2$ spin liquid. In either case the gap between the ground state and first excited state should go to zero for a torus geometry (for the Dirac SL because it is gapless and for the $\mathbb{Z}_2$ QSL because there should be a four-fold ground state degeneracy). A recent ED study has investigated $N = 36$, 42 and 48 site clusters [4]. The encountered gaps are $\Delta = 0.010$, 0.020 and 0.021, respectively. In other words, if we take the smallest ED gap for the KHAFM (the $N = 36$ site cluster), then we can fit the lowest lying state from every single momentum sector within this gap for our $N = 30$ and 32 site clusters. If we take the ED gap from the largest system size studied, the $N = 48$ site cluster, then for all of our clusters, $N = 18 - 32$ sites, we can fit the lowest lying state from every single momentum sector within this gap.

- Recent studies of Kalmeyer-Laughlin chiral spin liquids. Such phases have a two-fold degenerate ground state manifold (GSM) for a toroidal geometry which is exponentially split on finite sized systems, meaning the gap between the ground state and first excited state should scale to zero. As a particularly enlightening comparison we can compare our $N = 32$ site honeycomb results with results for CSLs on $N = 32$ site honeycomb lattices. Comparing to data from [5] and [6] they find gaps of $\Delta = 0.011$ and $\Delta = 0.035$ respectively. For our $N = 32$ site results we can again fit at least 16 states (one from each momentum sector) within these gaps.

In summary, when compared to other ED studies for states, which we either know to be gapless or know/suspect to have a ground state degeneracy, the gaps encountered in our study are exceptionally small, not just for the first excited state but for the lowest lying state in every single momentum sector.

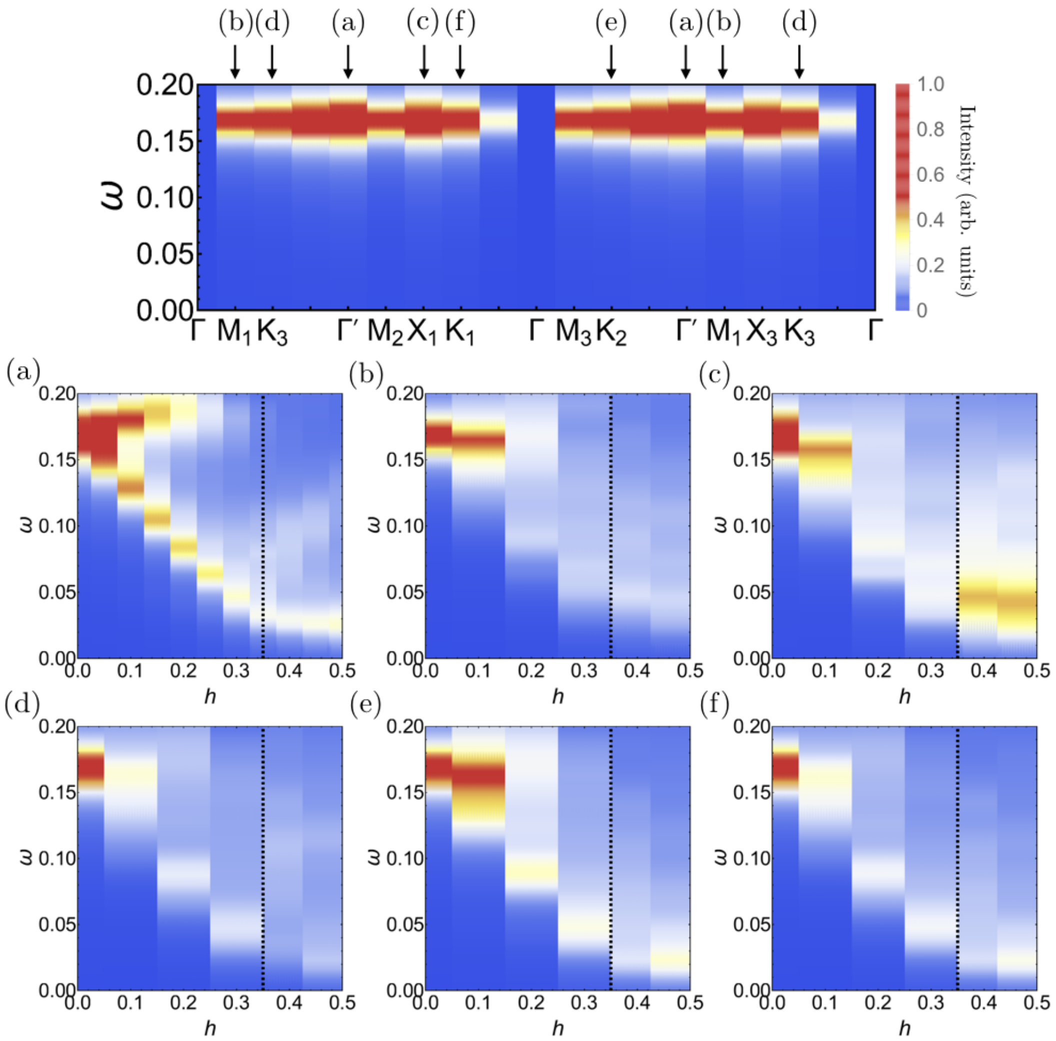

Supplementary Figure 4. Dynamical spin structure factor at various high-symmetry points. (a) Γ', (b) one of the M points, (c) one of the X points, and (d)-(f) the three distinct K points. The intensity is shown as a function of increasing field along the cut $\theta = 82.5°$, the upper of the two dashed red lines in Fig. 1(c) of the main text.

[1] M. A. Metlitski, D. F. Mross, S. Sachdev, and T. Senthil, Phys. Rev. B **91**, 115111 (2015).

[2] M. Hermanns, S. Trebst, and A. Rosch, Phys. Rev. Lett. **115**, 177205 (2015).

[3] J. Richter, J. Schulenburg, and A. Honecker, "Quantum magnetism in two dimensions: From semi-classical néel order to magnetic disorder," in *Quantum Magnetism*, edited by U. Schollwöck, J. Richter, D. J. J. Farnell, and R. F. Bishop (Springer Berlin Heidelberg, Berlin, Heidelberg, 2004) pp. 85–153.

[4] A. M. Läuchli, J. Sudan, and R. Moessner, Preprint at https://arxiv.org/abs/1611.06990 (2016).

[5] Y.-F. Wang, H. Yao, Z.-C. Gu, C.-D. Gong, and D. N. Sheng, Phys. Rev. Lett. **108**, 126805 (2012).

[6] C. Hickey, L. Cincio, Z. Papić, and A. Paramekanti, Phys. Rev. Lett. **116**, 137202 (2016).
